# Metabolomic approach to investigate Dactylis glomerata L. from the VIR collection

**DOI:** 10.18699/VJGB-23-16

**Published:** 2023-04

**Authors:** N.Yu. Malysheva, T.V. Shelenga, A.E. Solovyeva, A.E. Solovyeva, T.B. Nagiev, N.V. Kovaleva, L.L. Malyshev

**Affiliations:** Federal Research Center the N.I. Vavilov All-Russian Institute of Plant Genetic Resources (VIR), St. Petersburg, Russia; Federal Research Center the N.I. Vavilov All-Russian Institute of Plant Genetic Resources (VIR), St. Petersburg, Russia; Metabolomic approach to investigate Dactylis glomerata L. from the VIR collection; Federal Research Center the N.I. Vavilov All-Russian Institute of Plant Genetic Resources (VIR), St. Petersburg, Russia; Leningrad Research Agriculture Institute Branch of Russian Potato Research Centre, Leningrad region, Russia; Leningrad Research Agriculture Institute Branch of Russian Potato Research Centre, Leningrad region, Russia; Federal Research Center the N.I. Vavilov All-Russian Institute of Plant Genetic Resources (VIR), St. Petersburg, Russia

**Keywords:** Dactylis glomerata, genetic resources, metabolomic profiling, character polymorphism, Dactylis glomerata, генетические ресурсы, метаболомное профилирование, полиморфизм признаков

## Abstract

The perennial grass cocksfoot (Dactylis glomerata L.) is a valuable early highly nutritious crop used as green fodder in agricultural production. The species is widespread across the Eurasian continent; it is characterized by plasticity and high ecological and geographical variability. The article considers the metabolic profiles of 15 accessions of the cocksfoot from the collection of the N.I. Vavilov Institute of Plant Genetic Resources (VIR). The material is represented by varieties and wild forms of various origin: the European part of the Russian Federation, Norway and Finland. The study was carried out using gas-liquid chromatography coupled with mass spectrometry. The study and comparison of groups of metabolites of cocksfoot accessions of various ecological and geographical origin was carried out. Statistical processing included the calculation of the main parameters of variability, factor analysis of the correlation system (Q- and R-technique), cluster analysis by Ward’s method and discriminant analysis. The variability of the quantitative and qualitative composition of the substances identified was revealed. Based on statistical processing of the results obtained, five groups of cocksfoot accessions were identified, differing in the profile of metabolites. One of the groups with a similar composition of metabolites consisted of accessions from one ecological and geographical region; another, of accessions of different origin. Significant differences were noted in the metabolomic profiles of a late-maturing wild cocksfoot accession from the Republic of Karelia at the booting stage from early- and mid-maturing accessions at the heading stage; it contained the largest number of free amino acids and the smallest number of identified primary and secondary metabolites. Wild-growing accession k-44020 from Norway surpassed other wild-growing accessions in the content of free amino acids, sugars and phosphates at the heading stage. Wild-growing accessions differed from breeding varieties with a high content of proline and threonine, indicators of high resistance to lack of moisture and high air temperature.

## Abstract (ru)

Многолетний злак ежа сборная (Dactylis glomerata L.) – ценная ранняя высокопитательная культура, используемая в качестве зеленого корма в сельскохозяйственном производстве. Вид широко распространен на территории евразийского континента, характеризуется пластичностью и высокой эколого-географической изменчивостью. В статье рассмотрены метаболитные профили 15 образцов ежи сборной из коллекции Всероссийского института генетических ресурсов растений им. Н.И. Вавилова (ВИР). Материал представлен сортами и дикорастущими формами различного происхождения: европейская часть РФ, Норвегия и Финляндия. Исследование проводили с помощью газо-жидкостной хроматографии, сопряженной с масс-спектрометрией. Выполнено изучение и сравнение групп метаболитов образцов ежи сборной различного эколого-географического происхождения. Статистическая обработка включала вычисление основных параметров изменчивости, факторный анализ системы корреляций (Q- и R-техника), кластерный анализ по методу Варда и дискриминантный анализ. Выявлена изменчивость количественного и качественного состава идентифицированных веществ. На основе статистической обработки полученных результатов выделили пять групп образцов ежи, различающихся по профилю метаболитов. В одной группе с похожим составом метаболитов оказались образцы из одного эколого-географического региона, в другой – образцы различного происхождения. Отмечены значительные отличия метаболомных профилей позднеспелого дикорастущего образца ежи из Карелии в фазе выхода в трубку от ранне- и среднеспелых образцов в фазе колошения: он содержал наибольшее количество свободных аминокислот и наименьшее число выявленных первичных и вторичных метаболитов. Дикорастущий образец к-44020 из Норвегии в фазе колошения превзошел остальные дикорастущие образцы по содержанию свобод- ных аминокислот, сахаров и фосфатов. Дикорастущие образцы отличались от селекционных сортов высоким содержанием пролина и треонина, устойчивостью к недостатку влаги и высокой температуре воздуха

## Introduction

Dactylis glomerata L. is widely distributed in Eurasia and North Africa. This culture is the fourth most important forage crop in the world, due to high yield and stress factors resistance (Stewart, Ellison, 2011). It is the earliest hay-type fodder crop in Northern Europe. The world collection of the N.I. Vavilov Institute of Plant Genetic Resources (VIR) presents varieties and wild populations of D. glomerata from various ecological and geographical areas. The material is represented by the tetraploid subspecies D. glomerata subsp. glomerata (2n = 28) with a high level of genetic diversity (Last et al., 2013). The main criterions in fodder crops breeding are high productivity, intensity of regrowth, and resistance to abiotic stress factors (Tulinov et al., 2019). Quality characteristics are rarely taken into account (Yakovleva et al., 2015).

Plants are able to synthesize a huge number of compounds having a variety of functions. Investigation of individual characters of their quantitative and qualitative composition determines the economic using of the culture (Maslennikov et al., 2012, 2013). N.I. Vavilov Institute, has experience of using metabolomic profiling in studying plant genetic resources from the VIR collection (Shelenga et al., 2014). The biochemical composition of the cocksfoot has not been studied enough. The recently conducted study of D. glomerata growing on the Aeolian Islands (Italy) by M. Mandrone et al. (2022) confirmed the relevance of its evaluation as a promising pasture crop that yields a good harvest of green mass under stressful conditions (drought, low temperatures, low pH soils). In the countries of North America, Europe and Oceania, D. glomerata is effectively used to combat soil erosion, desertification, for restoration of green areas after fires and logging. The authors also note the lack of information about metabolomic studies of D. glomerata. The study of the diversity of D. glomerata genotypes from the collection of VIR reveals accessions with optimal feed properties: high values of organic acids, essential fatty and amino acids, monosaccharides, polyols (inositol and its isomers), phytosterols, low concentrations of anti-nutrients (raffinose). Also it reveals accessions in metabolomic profiles (MP) which were dominated by substances – factors of resistance to abiotic stress (FSS, free amino acids – precursors of phenylpropanoids: phenylalanine, tyrosine, tryptophan; pipecolic acid, oxyproline (a structural compound of extensin, which is part of the matrix of the plant cell wall) (Solovyeva et al., 2019), oligosaccharides, monoacylglycerols, galactinol, mannitol, glycosides) and can be used in programs for breeding new varieties resistant to environmental stresses, as well as varieties with improved feed (Rasmussen et al., 2012; Solovyeva et al., 2020).

The purpose of our research was D. glomerata metabolomic profiles evaluation to assess the biochemical variability of varieties and wild populations, degree of similarity, differences, and identify the promising sources for breeding.

## Materials and methods

The material for research was 15 cultivar and wild accessions of D. glomerata from the VIR collection zoned in different regions of the Russian Federation, Norway and Finland (Table). The green mass of 14 accessions was collected at the heading stage, one late-maturing accession – at the booting stage. Samples preparation, GC-MS analysis, results and processing were carried out according to the protocol in three analytical replications (Loskutov et al., 2020). Statistical data processing was performed using application the software package Statistica 12.0 and included calculation of the main parameters of variation – mean, standard error, minimum and maximum, upper and lower level of the confidence interval of the mean at p = 0.05 and coefficient of variation; correlation analysis; cluster analysis by Ward′s method and Q- and R-technique of the analysis of principal components and discriminant analysis

**Table 1.**
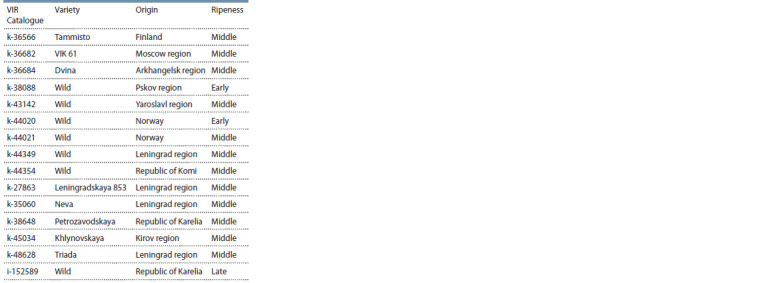
List of accessions of cocksfoot (Dactylis glomerata L.)

## Results and discussion

Cocksfoot green mass chemical composition

In total, 125 components from amino acids, organic acids, phenol-containing compounds, sugars, free fatty acids, polyols, glycosides, lactones, phosphates, sterols, and paraffins groups were identified.

Amino acids. The main nitrogenous substances of herbaceous plants are proteins, free amino acids and their amides, nucleic acids, nucleotides, and nitrogenous bases. Free amino acids are an important group of compounds involved in the synthesis of specific tissue proteins and other components necessary for organisms (Shkrobotko et al., 2009), contributing to maintaining the functional stability under stress conditions (Sampieva et al., 2010). Free amino acids, having a wide spectrum of pharmacological action, give other substances an easily digestible and harmless form, while enhancing their effect (Shilova et al., 2008). The green mass of the cocksfoot was found to contain 19 free amino acids, including six essential (valine, leucine, isoleucine, phenylalanine, tyrosine, tryptophan), and the nucleoside adenosine (Suppl. Material1). Nine of them (valine, alanine, leucine, isoleucine, glycine, threonine, serine, aspartic and glutamic acids and their derivatives – asparagine and glutamine; ornithine) were aliphatic; three (phenylalanine, tyrosine and tryptophan) – aromatic and two (proline and oxyproline) – heterocyclic amino acids. Phenylalanine, tyrosine and tryptophan are precursors of phenylpropanoids. Oxyproline is one of the main compounds of the cell matrix, the extensin protein, indirectly indicating the stress resistance of the accessions. Extensin is a glycoprotein with a high content of oxyproline and oligosaccharide side chains from arabinose. Pipecolic acid and proline also belong to the factors of plant protection from stress (Lotova, 2007; Solovyeva et al., 2019, 2020). A quite high content of pipecolic acid, which is related to non-protein amino acids, was detected. The predominant amino acids in the green mass of cocksfoot are oxyproline and glutamine (23.05 and 13.29 % of the total amino acids, respectively). The content of essential amino acids in the accessions is 19.65 %, where valine predominates (5.56 %). In combination with other BAS (biology active compounds: phenol-containing compounds (PhC), polysaccharides, organic acids (OA), macro- and microelements), it emphasizes the economic value of the green mass of cocksfoot and perspectives in breeding for improvement of feed quality. The total content of free amino acids varied from 91.77 to 346.08 conventional units (CU) (average 207.18). The highest values were determined in three accessions (more than 300 CU): Tammisto k-36566 (Finland), wild k-44020 (Norway) and wild i-152589 (the Republic of Karelia). The lowest were found in wild accession k-44349 (Leningrad region), high content of essential amino acids: k-48628 (59.30; Triada, Leningrad region), anti-stress factors: FSS precursor amino acids: i-152589 (25.14; wild, the Republic of Karelia) and k-44354 (24.10; wild, the Republic of Komi), pipecolic acid: k-27863 (27.58; Leningradskaya 853, Leningrad region), oxyproline: k-44354 (68.79; wild, the Republic of Komi; proline: k-44349 (24.27; wild, Leningrad region).

Supplementary Materials are available in the online version of the paper: https://vavilovj-icg.ru/download/pict-2023-27/appx2.pdf


Organic acids. Fruits and roots are characterized by the predominance of free OA; in grass, buds and leaves it is usually in the form of acidic salts. The most common types OA of aliphatic series are malic, citric, succinic, oxalic, phytic, acetic, tartaric, lactic, gallic and others. The value of OA in the diet is determined by their energy value and active participation in metabolism (Latypova et al., 2014). Up to 60 % of organic acids was malic acid (see Suppl. Material). In second place were inorganic phosphoric and fumaric acids, the content of which is 248.64 and 102.22 CU, respectively. The content of succinic, threonic, citric, ribonic, lactic, glyceric and ketogluconic acids varied in the range from 17.82 to 75.72 CU. The concentration of gluconic, oxalic, maleic, glucaric, erythronic, pyruvic, dehydroabietinic, azelaic, tartaric and aconitic acids did not exceed 10 CU, citraconic and methylmalonic acids – 0.11 CU. The use of green vegetable mass with a high content of malic, tartaric, citric, lactic and ascorbic acids in animal husbandry and poultry farming as the main feed or feed additive improves the absorption of nutrients, and also has antibacterial effect, which has a positive effect on the weight gain of farm birds and animals (Rasmussen et al., 2012; Solovyeva et al., 2019, 2020; Khan et al., 2022).

Lactone and phosphate forms of OA have been also identified. Lactone forms (erythrono-1,4-lactone, glucono- 1,4-lactone, glucono-1,5-lactone, on average 127.46 CU) are biologically active forms of organic acids, capable of binding heavy metals, protecting the cell from damage. The presence of phosphate forms (gluconic acid-6-phosphate, on average 2.71 CU) (see Suppl. Material) reflects the activity of metabolic processes in the plant (Cañete-Rodríguez et al., 2016). Data analysis shows a significant content of OA in the green mass of cocksfoot. On average, it was 1819.48 CU, depending on the variety; it varied from 1074.83 to 2579.87 CU (see Suppl. Material). The lowest values of OA were observed in wild accessions i-152589 (the Republic of Karelia) and k-38088 (Pskov region). The highest – in wild accessions k-44349 (2454.37; Leningrad region), k-44021 and k-44020 (2311.65, 2579.87; Norway), high content of malic: k-44349 (1667.06; wild, Leningrad region), k-27863 (1524.16; Leningradskaya 853, Leningrad region), tartaric: k-43142 (3.51; wild, Yaroslavl region) citric: k-44354 (115.68; wild, the Republic of Komi), k-35060 (101.77; Neva, Leningrad region), lactic acids: k-44349 (42.43; wild, Leningrad region), k-44354 (40.10; wild, the Republic of Komi), k-27863 (40.11; Leningradskaya 853, Leningrad region).

Phenol-containing compounds. PhC is one of the most numerous classes of natural compounds with biological activity. The intensity of their accumulation depends on stress factors, plant age and light conditions (Misin et al., 2010; Maslennikov et al., 2013). The accumulation of PhC is closely related to their function and development phase (Sazhina, Misin, 2011). It is noted that PhC have pronounced antibacterial activity, therefore, accessions of forage crops with a high content of PhC can be used not only to create new stress-resistant varieties, but also as an effective supplement to the daily diet in animal husbandry and poultry (Mahfuz et al., 2021). High values of caffeic acid in plant tissues contribute to protection against the penetration of fungal pathogens into them (Balmer et al., 2013). A total of 19 PhC were found in the green mass of cocksfoot (see Suppl. Material): free phenolcarboxylic acids (benzoic – 1.37, nicotinic – 0.55, 4-hydroxybenzoic – 0.65, protocatechuic – 2.20 and 2,3-dihydroxybenzoic – 0.09; average content – 4.86 CU), quinones (hydroquinone – 1.34, resorcinol – 1.19, pyrogallol – 3.04, and plumbagin – 1.01; average – 5.57), acyclic PhC (shikimic – 342,76 and quinic acids – 829,91; average content – 1172.67 CU) and phenylpropanoids (E)-4-hydroxycoric – 22.85, (E)-ferulic – 6.51, caffeic – 24.96, chlorogenic – 21.05, cryptochlorogenic – 4.26, neochlorogenic – 10.05 acids, coniferyl alcohol – 17.22 and α-tocopherol – 1.04; average – 106.99 CU). The predominant PhC were quinic and shikimic acids (64.28 and 26.55 % of the total PhC), which indicates the activity of the shikimate pathway of PhC synthesis, and may be associated with environmental stress impacts (Misin et al., 2010). The amount of PhC in cocksfoot on average was 1292.06 CU and varied from 165.27 to 1788.16 CU. In our study, the highest accumulation of PhC was in three accessions: varieties Leningradskaya 253 (k-27863), Tammisto (k-36566) and wild accession from Norway (k-44020) (1788.16, 1771.25, and 1783.67 CU), caffeic acid in Tammisto (45.26 CU; k-36566, Finland).

Carbohydrate composition. In the vegetative organs of forage grasses, the main products of photosynthesis are carbohydrates. Their nutritional value is determined by the amount of easily soluble carbohydrates – monosaccharides and sucrose. In our study, the total amount of sugars in cocksfoot averaged about 15 % of the dry mass, 71 % of which were represented by monosaccharides. Fifteen sugars were identified in the studied cocksfoot accessions: 11 monosaccharides, four oligosaccharides – three disaccharides and one trisaccharide (see Suppl. Material). The sugar content in cocksfoot averaged 4.00 (1.07–7.27) %. The majority of sugars were represented by monosaccharides – 2.85 (0.77–5.48) %, hexoses – 2.84 (0.76–5.47) %, pentoses – 0.014 %. Oligosaccharides were represented by disaccharides – sucrose, maltose and rutinose; trisaccharides – raffinose. The amount of oligosaccharides was 1.15 (0.30–1.78) %, where sucrose was 1.13 %. Metabolically active derivatives of sugars are lactone (glucose-1,4- lactone), phosphate (glucose-1-phosphate) and methyl forms (methylmannoside, methylpentafuranoside, methylglucofuranoside). The amount of sugar derivatives in cocksfoot was 366.2 (28.31–790.37) CU (see Suppl. Material). A number of sugars, such as glucose, sucrose, and raffinose, can accumulate under the influence of stress factors and reflect the activity of plant protection mechanisms from their effects. The nutritional value of feed is associated with a high sugar content, but raffinose has anti-nutritional properties (Solovyeva et al., 2019, 2020). The highest sugar content was determined in the wild accession k-44020 (7.27 %; Norway), monosaccharides (5.48 %), glucose (1755.90 CU), sucrose (1770.5 CU) in k-44349 (wild, Leningrad region), raffinose in k-43142 (27,18; wild, Yaroslavl region); the lowest – in the wild accession i-152589 (1.07 %; the Republic of Karelia), raffinose: k-48628 (2,09; Triada, Leningrad region).

Free fatty acids, acylglycerols and alkanes. The lipid complex of plants is represented by structural and reserve forms. Most of the lipids are found in the tissues of leaves and inflorescences; a lesser part is in the roots and stems of plants. During vegetation, the content of lipids decreases in the green mass, especially in the reproductive phase of development (Novikov, 2012). Eleven free fatty acids (FA) were identified in the green mass of cocksfoot: saturated (pelargonic, undecylic, palmitic, stearic, begenic, lignocerinic, cerotinic), unsaturated (oleic, linoleic, linolenic), hydroxyoctodecanoic acids and monoacylglycerols (MAG 1-C16:0; MAG 1-C18:0); and four alkanes (pentacosane, octacosane, nonacosane, hentriacontane) (see Suppl. Material). The high content of FA in the green mass of feed and feed additives has a positive effect on the growth and development of cattle (Shurson et al., 2015; Leiva, Granados-Chinchilla, 2020). The presence of monoacylglycerols and alkanes in plant tissues is associated with stress resistance (Solovyeva et al., 2019, 2020). The amount of free FA varied from 82.70 to 297.30 (on average 185.69 CU). Lipids of forage grasses have a lot of unsaturated FA 53 % of the total amount of FA, including 39 % essential, so the cocksfoot has a high nutritional value for livestock feeding. The amount of monoacylglycerols ranged from 8.82 to 19.37 CU (on average 14.43), alkanes – from 3.82 to 30.07 CU (10.86) (see Suppl. Material). A high accumulation of FA was observed in wild accessions k-38088 (297.30 CU; Pskov region) and k-44021 (256.78; Norway). Variety Tammisto (k-36566) was distinguished by the content of essential FA (126.56 CU), acylglycerols are in k-44354 (19.37; wild, the Republic of Komi), paraffins – in k-35060 (30.07; Neva, Leningrad region). The lowest FA values were observed in a wild accession from the Republic of Karelia (82.71 CU).

Polyols and phytosterols. Thirteen polyatomic alcohols were found in cocksfoot accessions. The range of variability of identified polyatomic alcohols varied from 119.58 to 269.02 CU (on average 179.87), most of them were related to sugar alcohols: glycerol, erythritol, trietol, xylitol, arabinitol, sorbitol, dulcitol, inositol (presented in three forms – chiroinositol, methylinositol and myo-inositol) and galactinol, their amount was 164.22 CU. The composition of alcohols also included amino alcohol (ethanolamine) and acyclic diterpene alcohol – phytol. The share of inositols was 25 % of the total amount of alcohols. Phytosterols (campesterol, stigmasterol, β-sitosterol) were detected as well – 34.09 CU (range from 15.92 to 53.70) (see Suppl. Material). Among phytosterols, β-sitosterol prevailed (24.57 CU). In addition, the phosphate forms of glycerol and inositol, and the products of glycerophospholipid metabolism (glycerol-5-phosphate, myoinositol- 2-phosphate, in total – 20.26 CU) were identified. A high content of phytosterols, the quantitative and qualitative composition of polyols characterizes not only the feed value of the green mass (inositol and its derivatives), but also resistance to stress factors (dulcitol, galaktinol) (Noiraud et al., 2001; Solovyeva et al., 2019, 2020). The study revealed accessions with high alcohol content: Petrozavodskaya (269.02) and Tammisto (247.04), wild accession k-44021 (256.75) from Norway, inositol and its derivatives are in k-43142 (114.10; wild, Yaroslavl region) and k-44020 (105.70; wild, Norway), dulcitol is in k-44020 (63.90; wild, Norway), phytosterols and galaktinol – in k-36682 (53.70 and 85.79; VIK 61, Moscow region).

Glycosides. Biologically active secondary metabolites of plants include glycosides, playing an important role in plant protecting and interacting with other organisms. Antirrhinoside and its derivatives are iridoids. They protect the plant from pathogens and pest insects: they repel leaf-eating and nonpollinating insects. Derivative of antirrhinoside, antirride, has antimicrobial and fungicidal activity (Matveeva, Sokornova, 2017). Lupeol (triterpenoid) has an estrogenic, androgynous, antimicrobial, and anticancer effect, and is used as a chemotherapy drug for a number of diseases (Gallo, Sarachine, 2009). Five glycosides were found: methylpentofuranoside, methylmannoside, methylglucofuranoside, antirrhinoside and lupeol (see Suppl. Material). The first three glycosides were discussed earlier in the section “Carbohydrate composition”. Antirrhinoside and lupeol were not found in all the studied accessions. The maximum amount of antirrhinoside was found in variety Leningradskaya 853 (k-27863; 63.48 CU), lupeol – in wild accession k-38088 from Pskov region (14.69 CU).

Variability of metabolome in the studied accessions of cocksfoot

This study revealed significant variability in the metabolomic profiles of D. glomerate L. accessions. In the course of factor analysis of the correlation matrix, 13 factors were identified, covering a total of 99.3 % of variability. First four factors provide 70.6 % variability, the other nine, only 28.7 %. Factor 1 (27.4 % variance) correlates with the content of 48 out of 126 compounds: 17 with an average (0.49 > D > 0.25) and 31 with a high degree of determination (D ≥ 0.50), where coefficient of determination D = r2, and r is the loading of character on the axis. According to this factor 12 PhC, 4 phosphates, 2 lactones, 2 sterols vary. Factor 2 (15.4 %) determines the variability of 26 compounds: 13 with an average and 13 with a high degree of determination. It is associated with the variability of 13 amino acids. By factor 3 (12.7 %), the content of 19 compounds varies (5 with an average and 14 with a high degree of determination). By this factor varies the content of 6 fatty acids and urea. Factor 4 (15.3 %) is associated with the variability of the content of 28 compounds: 13 with an average and 15 with a high degree of determination. The largest number of compounds that vary by this factor are OA (9) and sugars (7). The following nine factors are associated with the variation of a limited number of compounds. Factor 5 (4.8 %) is strongly correlated with H-quinone, nonacosan and pentacosane and glycerol. Factor 6 (5.3 %) is associated with variation of the amino acid tyrosine, ribose, altrose, sorbose and galactose sugars, and DHO-benzoic PhC. Factor 7 (3.0 %) is associated with the variation of alcohol trietol, alkane hentriacontane and methylpentofuranoside glycoside. Methylphosphate, dulcitol, methyl-inositol, and sterol β-stigmasterol vary by factor 8 (3.8 %). Factor 9 (4.1 %) causes variation of citric OA and pelargonic FA. Factor 10 (2.0 %) determines the variation of non-protein pipecolic amino acid, glycoside methylglucofuranoside, myo-inozitol-2-phosphate, linoleic FA and PhC ferulic acid. Variation of the octacosane alkane and the me-malonic OA occurs by factor 11 (2.7 %). Factor 12 (1.8 %) determines the variability of the alcohol xylitol. The variation in the content of all metabolites is poorly related to factor 13 (1.1 %). Some compounds vary by two factors.

**Fig. 1.**
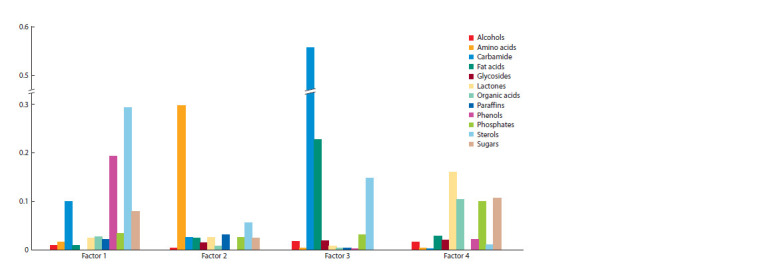
Average determination of groups of metabolites by the first four principal components of variation.

Thus, in the system of inter-population correlations between metabolites, four large pleiades of traits are distinguished (Fig. 1). The first pleiad is related primarily to the variation in the content of phenols and sterols; the second describes the variation in the content of amino acids, the third – fatty acids and urea; the fourth – lactones, organic acids and saccharides. Another eight factors describe the variability of relatively independent traits that are poorly correlated with traits from the main pleiades

**Fig. 2.**
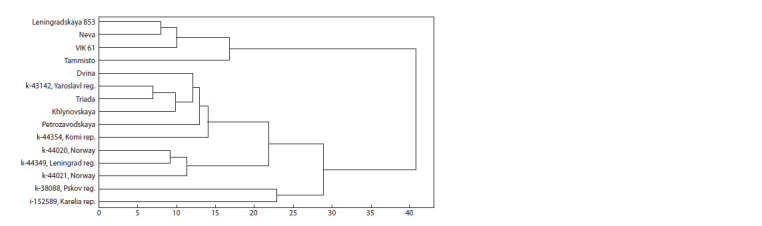
Classification of cocksfoot accessions by the content of metabolites (cluster analysis, Ward’s method).

When using the Q-technique of factor analysis, only two groups of accessions are allocated: wild accession i-152589 from the Republic of Karelia (Factor 2) and all other accessions (Factor 1). The Ward′s method was used for the cluster analysis procedure. Based on the results of cluster analysis of metabolic profiles, five groups of accessions characterized by similar metabolomic profiles were identified: wild accession from Pskov region (k-38088); wild accession from the Republic of Karelia (i-152589); wild accessions from Norway (k-44020) and (k-44021) and Leningrad region (k-44349); varieties Dvina, Khlynovskaya, Petrozavodskaya, Triada and wild accessions from Yaroslavl region (k-43142) and the Republic of Komi (k-44354); varieties Leningradskaya 853, Neva, Tammisto and VIK 61 (Fig. 2).

**Fig. 3.**
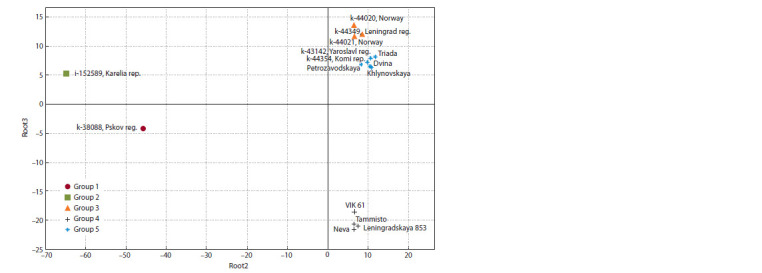
Differentiation of cocksfoot accessions by the content of metabolites (general discriminant analysis).

The group affiliation of the studied accessions of cocksfoot has a significant effect on the content of 100 metabolites out of 136 identified, i. e. the features of the MP of each of the groups. According to the results of the classical discriminant analysis, “information value”, the following components reliably distinguishing the groups were identified: lupeol, monosaccharides, arabinose, raffinose, ethanolamine, erythritol, arabinitol. Four variables were identified that ensure the separation of accessions taken in the study: Root1 (arabinitol, ethanolamine), Root2 (arabinose, erythritol), Root3 (sum of monosaccharides), Root4 (sum of monosaccharides, arabinitol). The most obvious separation of accessions was obtained in the Root2 and Root3 axes (Fig. 3).

The first group is characterized by a high content of fatty acids, PhC and polyatomic alcohols, and a low content of glycosides; the second group – by a high content of most amino acids and a low content of sugars and sterols. The third group, consisting of three wild accessions, is characterized by an increased content of organic acids and sugars. Earlymaturing accession from Norway (k-44020) also showed a high content of free amino acids, as well as a late-maturing wild accession from the Republic of Karelia (i-152589). The first one attracts attention as material for creating late-maturing variety, herbal mixes with legumes. Its cutting ripeness occurs during the budding of clover and alfalfa in the North-West of the Russian Federation. These three groups of wild cocksfoot are characterized by a high content of proline and threonine, amino acids that are associated with resistance to stress in response to adverse abiotic factors (Ibragimova et al., 2010; Pandyan et al., 2018).

Wild cocksfoot accessions showed a higher resistance and responded to this stressful situation by accumulating proline. On the other hand, a lesser proline and threonine accumulation and lesser resistance to drought of varieties is a consequence of the process of “domestication”, when the selection in the population was carried out only for economically valuable traits. In this case, the resistance of varieties to stress factors may decrease. The fourth group is characterized by average values of the content of most compounds. Varieties Dvina and Petrozavodskaya created from local wild populations; variety Khlynovskaya – by “free-limited” cross-pollination of local Dedinovskaya from Moscow region. Wild accessions from this group are from the Republic of Komi (k-44354) and Yaroslavl region (k-43142). In this case, it is impossible to explain the grouping of accessions from geographically remote locations into one group. In the fifth group, consisting exclusively of selective varieties, there is a high content of sugars and PhC and a low content of phosphates. Two varieties from the fifth group (Leningradskaya 853 and Neva) were derived from wild populations of cocksfoot from Leningrad region, VIK 61 – by re-pollination of a wild accession from the foothills of the Caucasus with wild accessions from the non-Chernozem zone; the pedigree of variety Tammisto from Finland is unknown. In this group, there were only two varieties originating from one common region. A generalized discriminant analysis model was used to evaluate the degree of differentiation of the selected groups of accessions by metabolomic profiles. The final discriminant functions included nine indicators: the content of lupeol, erythrono-1,4-lactone, glucono-1,4-lactone, methylmalonic acid, pyrogallol, glucosamine, maltose, ethanolamine, and the sum of PhC. The predicted classification based on the constructed functions gives 100 % correct solutions. Thus, the proposed hypothesis about the similarity of metabolomic sections in accessions from a common territory and having similar genotypes is only partially confirmed.

The study of the metabolomic profiles of this culture has been scarcely carried out, as was noted in the work devoted to the study of the features of MP of D. glomerata, conducted by M. Mandrone et al. The researchers noted the importance of studying MP to identify the effectiveness of the response of a plant organism to environmental stress, as well as phylogenetic features of culture. The association of high concentrations of valine, asparagine, phenylalanine, fumaric acid and PhC with stressful growth conditions of D. glomerata, in particular with drought and increased salt content in the soil of volcanic rocks and the presence of volcanic gases, was noted (Mandrone et al., 2022). The comparison of the data obtained by Italian researchers with our results is rather conditional, since other research methods were used in the work of M. Mandrone et al.: UHPLC−MS, NMR analysis and spectorophotometry. However, they established the prevalence of fumaric acid in the MP of D. glomerata, which coincides with our data. The researchers also stressed that increased stress exposure leads to an increase in the accumulation of PhC. S. Rasmussen et al. (2012) noted that there is an increase in quinic and shikimic acid, phytosterols and raffinose in the MP forms of Lolium perenne, resistant to drought. In the current study, quinic and shikimic acids were established as dominant in the group of PhC of MP. Accessions of D. glomerata with the highest concentration of phytosterols, raffinose and quinic and shikimic acid were identified as potentially resistant to stress. In the article of D. Balmer et al. (2013), it is shown that high values of caffeic acid in the tissues of cereal crops protect the plant from fungal pathogens. That was taken into account when we distinguished economically significant D. glomerata accessions. The same compounds dominate among organic acids, oligosaccharides, phytosterols and PhC in the MP of cocksfoot and oat seedlings and green mass of peavine previously studied by us (Solovyeva et al., 2019, 2020; Loskutov et al., 2021). In the MP of oat and cocksfoot seedlings in the group of polyols and FA, the main substances are isomers of inositol and linoleic and palmitic acids, peavine and cocksfoot – glycosides: methylglucoside (Loskutov et al., 2021). An iridoid glycoside – antirrhinoside was detected in both the green mass of peavine and cocksfoot (Solovyeva et al., 2019, 2020). There are significant differences in the qualitative composition of the other groups. These differences in MP of different cultures make it possible to assert that MP reflects the specific features of a culture.

## Conclusion

As a result of the study, new data on the qualitative and quantitative composition of MP of D. glomerata was obtained. With the help of discriminant analysis, the most significant indicators of the MP of the green mass of D. glomerata were established. Accessions combining feed value with stability indicators were identified (i-152589, k-27863, 35060, 36566, 43142, 44020, 44349, 44354), as well as those with high indicators of feed value (k-38088, 38648, 44021, 48628) and anti-stress factors (k-27863, 36682, 38088), suitable for breeding highly nutritious varieties resistant to abiotic factors. The study confirms the potential of D. glomerata as a promising forage crop. We have confirmed that the optimal plant stage for feeding animals is the stage of heading, when the content of nutrients is high and at the same time the stems of plants are not yet coarsened. But additional research is required to identify changes in metabolites at different stages of cocksfoot’s life cycle.

## Conflict of interest

The authors declare no conflict of interest.
